# Co-Design of a Virtual Reality Multiplayer Adventure Game for Adolescents With Autism Spectrum Disorder: Mixed Methods Study

**DOI:** 10.2196/51719

**Published:** 2023-12-08

**Authors:** Silvia Gabrielli, Melanie Cristofolini, Marco Dianti, Gianpaolo Alvari, Ersilia Vallefuoco, Arianna Bentenuto, Paola Venuti, Oscar Mayora Ibarra, Elio Salvadori

**Affiliations:** 1 Digital Health Research Fondazione Bruno Kessler Trento Italy; 2 ODFLab - Observational, Diagnosis and Education Lab Department of Psychology and Cognitive Science University of Trento Trento Italy; 3 Meeva Srl Trento Italy; 4 Cooperativa Socio Sanitaria Albero Blu Trento Italy

**Keywords:** co-design, virtual reality environments, autism, social skills interventions, multiplayer game design, serious games

## Abstract

**Background:**

Virtual reality (VR) adventure games can offer ideal technological solutions for training social skills in adolescents with autism spectrum disorder (ASD), leveraging their support for multisensory and multiplayer interactions over distance, which may lower barriers to training access and increase user motivation. However, the design of VR-based game environments for social skills training is still understudied and deserves the deployment of an inclusive design approach to ensure its acceptability by target users.

**Objective:**

We aimed to present the inclusive design process that we had followed to develop the Zentastic VR adventure game to foster social skills training in adolescents with ASD and to investigate its feasibility as a training environment for adolescents.

**Methods:**

The VR game supports multiplayer training sessions involving small groups of adolescents and their therapists, who act as facilitators. Adolescents with ASD and their therapists were involved in the design and in an explorative acceptability study of an initial prototype of the gaming environment, as well as in a later feasibility multisession evaluation of the VR game final release.

**Results:**

The feasibility study demonstrated good acceptability of the VR game by adolescents and an enhancement of their social skills from baseline to posttraining.

**Conclusions:**

The findings provide preliminary evidence of the benefits that VR-based games can bring to the training of adolescents with ASD and, potentially, other neurodevelopmental disorders.

## Introduction

### Background

Impairments in social skills represent a central feature of autism spectrum disorder (ASD), a neurodevelopmental disorder affecting verbal and nonverbal social communication in children [1]. Approximately 44% of children with ASD have IQ scores in the average and above-average range [2]. However, they may experience difficulties in social skills such as an absence of social reciprocity, a poor display of eye contact and facial expressions, a lack of nonverbal behaviors and gestures, and difficulty in acquiring and maintaining peer relationships. Individuals with ASD commonly have difficulties with exhibiting the appropriate social skills across contexts [3] and facing social tasks required by complex situations in community life, school, and work [4].

Different methods and interventions have been designed to facilitate the acquisition of social skills in adolescents with ASD, including video-based instruction, in vivo instruction, high-tech augmentative and alternative communication, low-tech augmentative and alternative communication, behavioral skills training, social skills training [5], and play-based interventions in virtual environments.

Virtual reality (VR) is a computer-generated 3D simulation of imaginary or real-life environments that is explored and interacted with by real-time users. VR can take variant forms, ranging from desktop computer rendering of a highly interactive virtual world to a fully immersive multisensory environment in laboratories [6]. VR has some intrinsic appeal as a training tool for children with ASD, who tend to learn using visual support [7,8]. A VR-based learning environment allows participants to practice social skills in a nonthreatening, synthetic, but realistic, motivating, controllable, and diversifiable environment during learning [9]. There is evidence that VR technologies can, and do, represent authentic and plausible scenarios and social encounters, thereby reflecting and supporting real-world conventions, understandings, and behaviors for a range of different user groups [10-13]. Supporting multisensory interactions for multiple users over distance is another salient feature of VR that makes social skills training accessible in diverse formal and informal learning settings [14].

Despite its promise, the VR-based learning environment is still understudied and warrants further development and testing. Early efforts on studying VR-based learning for individuals with ASD proceeded from embedding multimedia direct instruction in a virtual world [15] to emphasizing multiplayer simulation and scenario-based social role-play [7,16,17]. In a recent pretest-posttest study with 30 children with ASD [17], researchers examined the effects of desktop VR–based social cognition training that involved children in social role-play with 2 to 3 peer- and clinician-controlled, confederate avatars in daily life social scenarios. The study showed participants’ improvements in measures of emotion recognition, social attribution, and executive function of analogical reasoning. Another explorative study [18] provided preliminary evidence on the positive effect of desktop VR–based play- and design-oriented social skills training for children with ASD. The study also indicated some main challenges in designing and implementing a comprehensive social skill intervention for learners with ASD, such as the need for a longer duration of practice and learning sessions for novel or demanding social tasks. Previous work has also observed that gamified therapeutic interventions may run the risk of having a reduced appeal for children, especially when marginal relevance is given to game design, neglecting children’s interests and their experience with video games available in the market [19]. According to recent studies, such risk is particularly relevant because children with ASD tend to spend nearly twice the amount of time playing video games than typically developing children do [20]. In a recent study, the authors proposed an inclusive design model to guide the development of effective and enjoyable therapeutic games, stressing the need to integrate knowledge from different perspectives, typically those of clinical experts, designers, and children [19].

### Objective

The purpose of this study was to present how we applied an inclusive co-design approach [21,22] to develop a VR adventure game named Zentastic, fostering social skills acquisition in adolescents with ASD.

## Methods

### Overview

We describe our work in eliciting requirements from therapists and adolescents and merging their contributions in an early prototype of the multiplayer game; preliminary testing of the game with adolescents was performed to uncover their acceptance of the game, feedback, and preferences with respect to the game scenarios and mechanics proposed. We also describe how we incorporated the results of this preliminary explorative phase into the final release of the VR game and how we further evaluated the feasibility of our intervention with 12 adolescents and their therapists over a period of 3 weeks. Finally, in the Discussion section, we report the promising results achieved in the study, suggesting VR-based gaming interventions as viable and motivating solutions for training social skills in adolescents with ASD.

### Co-Design of the Zentastic VR Gaming Intervention

This section describes the inclusive co-design approach [19,21] applied to develop the Zentastic VR gaming intervention by leveraging on the ideas and suggestions of the designers, the neurodevelopmental psychologists, and the children with ASD.

#### The Game Design Context

The Zentastic VR adventure game was designed in the context of the European Project XR4A (extended reality–enabled social skills therapy for adolescents with autism and neurodevelopmental disorders), which aimed at experimenting the application of VR gaming technologies as potentially engaging and motivating environments for training of social skills in adolescents with ASD. The game was developed through a close collaboration with ASD experts at ODFLab (Observational, Diagnosis, and Education Lab, University of Trento), a specialized center for children and adolescents with ASD and neurodevelopmental disorders (Cooperativa Albero Blu) in Italy.

The motivation for developing a VR game to engage and train social skills in adolescents with ASD started with the assumption that VR technology can be conceptualized as a mediator of social interaction [19]. It can be an instrument for scaffolding and facilitating interpersonal relationships between adolescents, their therapists, and their peers in a training and therapeutic context [23]. To achieve this, it is important to design game experiences that are sufficiently engaging and valuable, so that the adolescents would feel motivated to initiate social interaction and communication in the playing setting, as well as to generalize the social skills acquired in everyday settings.

#### Eliciting Requirements From Experts

Requirements from psychologists and therapists at the local autism center, Cooperativa Albero Blu, were aimed at defining the structure of the VR game, the objectives, and the roles of players. To define these aspects, 4 meetings involving both designers and experts were arranged during the initial stage of the design process [19,21]. Each meeting lasted for almost 2 hours and involved between 2 and 4 experts from the local autism center. The first meeting focused on (1) introducing the 2 teams, interaction, VR game designers and ASD experts, (2) explaining the project objectives, (3) presenting state-of-the-art and key examples of VR game solutions for adolescents with ASD, and (4) sharing ideas and previous experiences of the teams in co-design methodologies [19,21,22] to be deployed in the VR game design.

The following meetings were arranged as brainstorming sessions aimed at establishing the conditions for cocreation through a merging of design ideas and clinical requirements. For this purpose, after each meeting, the designer team produced a document summarizing the main ideas that emerged and the decisions taken and sketching some initial proposals for the game structure to be discussed in the following meeting.

During the subsequent phases of the VR game design and prototyping, additional meetings were organized to evaluate our progress. The analysis of the requirements collected from the ASD experts and the study of their current use of game-based approaches in the training of adolescents with ASD allowed us to properly frame the scope of the VR gaming intervention as well as our target user group.

As the main goal of the project was to support social skills training in adolescents with ASD as an important enhancement in their daily activity and future lives, the target users specified by experts were adolescents aged 13 to 18 years with level 1 ASD severity, according to the *Diagnostic and Statistical Manual of Mental Disorders*, 5th edition [1]. Children with level 1 ASD are those with higher cognitive development who have more chances of having an autonomous life if adequately assisted through their childhood and adolescence [1]. Therefore, we concentrated our efforts on this target user group, which might be more suitable and motivated to engage with multiplayer VR games and train their social skills by interacting with peers and therapists. The inclusion criteria defined by our experts were (1) a minimum IQ level of 70, which refers to the ability to understand the environment and hence, understand the VR game challenges presented; and (2) a diagnosis of autism with an Autism Diagnostic Observation Schedule module 3 with a minimum severity level of 4 (ie, having a fluent verbal capacity).

The game intervention plan recommended by the experts was characterized by a progressive scaffolding and elicitation of social interaction in the VR game, which is aligned with current practices in traditional treatment. This plan ensured that adolescents first familiarized themselves with the play environment, were introduced to the colocated play goals and challenges by their therapist, and then they shared the play experience with their peers (in groups of 2-4 players per session) to meet the game objectives. The VR game should enable social skills training over multiple sessions; therefore, the experts also expressed the need for having the possibility of tuning the level of difficulty of the game challenges to better adapt them to the players’ needs and familiarity with the VR game.

The initial game structure was derived from the experts’ requirements, defined around the Zentastic adventure game story. In the game, players were asked to enroll on a space journey to the Zentastic planet. The game mission consisted of overcoming different challenges in the attempt to find and retrieve the precious Zennite material, which was needed by the scientists on Earth to repair the ozone layer and thus ensure the survival of Earth.

The players’ interaction patterns recommended by experts were based on a cooperative and collaborative model for the game experience, leveraging synergies between different user abilities, shared goals, and complementarities of player actions [24].

The experts also defined a set of core behavioral skills required in the game to foster social interaction and social skills training in adolescents with ASD. The required social skills were (1) turn taking; (2) social interaction and cooperation; and (3) executive functions such as sustained attention, selective attention, and inhibition that strengthen social skills.

These requirements led to the design of a basic structure of game mechanics, as reported in Table 1, to be implemented in the first VR game prototype and tested with adolescents for subsequent refinement according to their preferences and feedback.

**Table 1:** Core behavioral skills and game mechanics that were defined by experts in the design phase.

Game session and objectives		Game mechanics
**Introduction**		
	Sustained attention	The player has to pay attention to the instruction provided by the facilitator.
**First mission (Coin Hunt)**		
	Selective attention and inhibition	The player has to discriminate between the target objects and nontarget objects and avoid picking up nontarget objects.
	Cooperation	The player has to collaborate with other players to collect as many target objects as possible.
**Second mission (Space Station)**		
	Social interaction	The player has to interact with an agent to obtain a target object.
	Turn taking	The player has to respect his turn to obtain a target object.
**Third mission (Planet Battle)**		
	Sustained attention and inhibition	The player has to discriminate between the target objects and nontarget objects.
	Cooperation	The player has to collaborate with other players to obtain the target objects as fast as possible.

### The Zentastic Multiplayer VR Adventure Game

The Zentastic adventure game is an immersive multiplayer VR game (developed in Unity and deployed on Meta Oculus technology) aimed at promoting social skills training in adolescents with ASD. The game is designed to be played by small groups of adolescents (2-4 players per session) in the presence of a therapist who acts as a facilitator in the virtual environment. The overall objective of the adventure game is to go through different missions of the Zentastic journey, facing and overcoming the challenges presented to achieve the final goal of collecting the Zennite material and taking it back to Earth to save the planet. At the start of the game session, players gathered in front of the fire in a common room, where they were introduced to the game plot and objectives. The players were then presented with the first mission called the Coin Hunt, where each player needed to collect a target number of coins (level of difficulty set by the facilitator) of a certain color hidden in a park to proceed with the following mission. The targeted behaviors related to social interaction trained by this mission are sustained attention, selective attention, and inhibition required to collect only the right coins during their hunt. In the Space Station mission, the players were asked to interact with a ticket-office agent (role played by the facilitator) to buy a ticket for the space shuttle that would take them to the Zentastic planet. Here, the targeted behavior trained is mainly turn taking, as the players would have to respect their turn while queuing to buy the ticket and initiate social interaction with the ticket agent to purchase the ticket. In the Planet Battle mission, the players had to climb a hill where the Zennite material was stored, and they were assigned colored laser guns to shoot colored stones falling down the hill that prevented them from reaching the Zennite. The targeted behaviors trained here are not only sustained attention, selective attention, and inhibition (to shoot the right stones) but also collaboration to reach the hill’s top as fast as possible as a team. At the end of the game, the players gathered again in the common room for a debriefing, congratulating on the experience and closing the session.

Each mission in Zentastic was designed to address a targeted behavior related to social interaction, with increasing complexity levels. A description of each mission and complexity level, corresponding to the intervention plan previously described, is provided in Textbox 1.

**Brief description of the Zentastic game missions and their challenges.**

**First mission (Coin Hunt)**

- In this mission, the player has to collect a target number of coins that are each assigned a color and hidden in a park. The target number is set by the facilitator (the level of difficulty can vary), and players have to achieve the target number of coins as a team. The team score increases or decreases according to each right or wrong coin collected. An additional bonus score is added if the players accomplish their individual targets within certain time thresholds. Players can help each other during the game to find the coins. As soon as the challenge is completed, multimedia effects appear, and a gold coin is assigned to each player as a reward in the gazebo.

**Second mission (Space Station)**

- Here, the players have to buy the ticket to get into the shuttle that will take them to the Zentastic planet. Players need to interact with a ticket agent (played by the facilitator) who may have different conversational attitudes (supportive vs unsupportive) toward the player (to vary the level of difficulty). When reaching the ticket counter room, each player has to respect their turn to talk with the ticket agent by waiting in a colored spot (previously assigned). A happy meter will increase every 3 s if players stay within their spot and decrease if they leave their spot and buzz around.

**Third mission (Planet Battle)**

- Here, players are on the Zentastic planet, climbing a hill to obtain the Zennite material. To reach the hill’s top, each player has to shoot at the rocks of assigned color that are challenging their climbing. Shooting at the wrong rock means losing 1 life. When all lives are lost, the player has to restart climbing from the bottom of the slope. The facilitator can vary the level of difficulty either by assigning the number of lives available to each player or by varying the speed of the falling rocks. Once the players have completed the challenge, the Zennite pile explodes, and each player gets a piece of Zennite to bring back to Earth.

### Uncovering Adolescents’ Expectations and Preferences

An initial evaluation of an early prototype of the Zentastic VR adventure game was conducted with children and adolescents with ASD who visited the autism center to investigate the acceptability and level of engagement of the target users with the game intervention designed. This explorative phase of co-design [21,22] was focused on collecting adolescents’ expectations and preferences regarding the game experience to feed this information into a refinement and finalization of our VR prototype. The methods used to collect the users’ feedback are as follows:

- Field observations of the behaviors exhibited by the participants during the VR game sessions to identify possible usability issues
- Short, structured postsession interviews aimed at understanding the level of satisfaction with the game experience and suggestions for improvements (Multimedia Appendix 1)

Overall, 31 participants with ASD were involved in this explorative study. This is justified in the literature review of studies using innovative solutions for adolescents with ASD by the difficulty of obtaining large samples when the aim of the study is related to psychopathology [25,26]. Participants included 25 (81%) adolescent boys and 6 (19%) adolescent girls; their average age was 13.2 (SD 3.41) years, their average Autism Diagnostic Observation Schedule score was 6.3 (SD 1), and their mean IQ was 96.6 (SD 20). All 31 participants performed at least 1 session of the VR game; 27 (87%) participants performed 2 sessions; 19 (61%) participants were involved in 3 sessions; and 3 (10%) participants performed 4 sessions.

Most participants (25/31, 81%) had an overall positive experience with the VR gaming environment, as evidenced by the high average scores (scale 1-5: 1=not at all; 5=very much) in the level of engagement and enjoyment with the VR experience (Table 2).

**Table 2:** Participants’ appreciation of the virtual reality (VR) gaming environment on a scale of 1 to 5 (1=not at all; 5=very much) in the explorative study.

Factor measured	Score, mean (SD)
Clarity of VR game	4.1 (0.8)
Engagement	4.1 (0.9)
Interaction with objects	4.3 (0.7)
Movement in VR	4.1 (0.9)
Object grasping	4.1 (0.9)
Interaction with the environment	4.2 (0.8)
Focus on activity	3.8 (0.9)
Enjoyment	4.5 (0.9)
Willingness to play again	4.6 (0.9)

Regarding the interaction with the VR hardware required for playing, some difficulties were observed, especially with the use of the controllers, and a few participants (4/31, 13%) experienced nausea after prolonged activity in VR (Table 3).

Regarding the emotional feelings of the participants after interacting with the VR gaming environment, 81% (25/31) of the participants reported feeling happy or relaxed, and 19% (6/31) reported feeling frustrated or confused.

From the postsession interviews with participants, some suggestions for improving the VR gaming experience were collected, which are as follows:

- Improving movement in VR: some participants pointed out that moving in the VR game was somehow too slow, while they preferred the teleporting functions.
- Making the VR scenarios more coherent with the story: some participants asked for improvement in the coherence of the 3 VR missions’ environments with the space adventure game plot by introducing relevant objects and characters in each of the game missions.
- Improving engagement and game dynamics in the VR game: most participants wanted to be provided with more engaging challenges in the game missions by including more interactive objects and characters, brighter colors, relevant music and sounds, and clear feedback to support progress toward the achievement of the game objectives.

Following stakeholders’ indications (including both the facilitators and the adolescents involved in the explorative study), improvements were implemented in the final release of the Zentastic game; for example, the 3 game missions were enriched by improving their interactive quality and overall coherence to the space challenge. We also designed and integrated a clearer scoring system within the game missions to provide feedback to participants during the game and facilitate an easier functional assessment of the players’ performance and targeted behavior by therapists during and after the game session.

We added specific controls for the facilitator in the VR game, allowing for a smoother personalization of the playing experience according to the level of complexity and ensuring the engagement of participants over multiple sessions in the VR game.

However, as the user sample used for this explorative study only partially fits the age of the target user group and the evaluation was based on an early prototype of the game, it was necessary to carry out an additional study with adolescents with ASD (aged 13-18 years) to evaluate the suitability of the experience. Nonetheless, such evaluation allowed us to ensure that our design was sufficiently usable and engaging for adolescents to support a multisession gaming intervention. This aspect is particularly relevant when designing for adolescents with ASD as VR game experiences not fitting their expectations and preferences may drastically reduce their motivation to engage with such experiences, as well as impair the overall effectiveness of such training interventions.

**Table 3:** Participants’ difficulties experienced with the virtual reality gaming environment on a scale of 1 to 5 (1=not at all; 5=very much) in the explorative study.

Factor measured	Score, mean (SD)
Difficulty with Oculus headset	2.3 (1.2)
Difficulties with controllers	2.5 (1.4)
Nausea	1.7 (1.1)

### Mixed Methods Study: Participants and Method

This section presents the study conducted to evaluate the feasibility of our VR game for training social skills in adolescents with ASD and the main results achieved.

We conducted an observational study on the interaction of 12 boys with ASD [20] (mean age 13.9, SD 3.1 years) with the final version of the Zentastic VR game. All participants could use the computer and internet and had prior experience of playing digital games (see Textbox 2 for inclusion criteria). The goals of the study were to evaluate the acceptance and enjoyment of the game [19,21] and to investigate whether it facilitated the targeted behaviors contributing to enhancing adolescents’ social skills.

The study was carried out at the Cooperativa Albero Blu Center premises for >1 month.

**Inclusion criteria to select participants.**

**The participants must**

- be aged between 13 and 18 years
- be diagnosed as having autism spectrum disorder (ASD; without intellectual impairment) using standard instruments from the Diagnostic and Statistical Manual of Mental Disorders-5 criteria, with Autism Diagnostic Observation Schedule module 3 having a severity level of at least 4 (within the ASD range spectrum)
- have a cognitive capacity of >70 as measured by the standard Wechsler Intelligence Scale for Children (fourth edition) [27]
- sign the consent form to participate in the study (applies to both the parent or tutor and the adolescent)

The participants played the VR game in groups of 2 to 4 players per session, assisted by the facilitator. Each adolescent participated in 3 sessions, scheduled weekly.

Each session lasted for 45 minutes, during which adolescents were first introduced to the game by the facilitator. Before the study launch, the researchers provided the facilitators with a dedicated training session on the use of the VR game controls and a prescripted document of guidelines to follow during the game sessions to ensure a consistent deployment of the game intervention among the different facilitators involved.

The methods used to assess the feasibility of the VR game were as follows:

- Participants’ score on the Social Skills Questionnaire (SSQ [28]), Teacher form, filled out by their therapist before and after the intervention in VR
- Postsession observations on the targeted behaviors exhibited by adolescents during the VR game to assess the effect of the game on training social skills
- An ad hoc user experience questionnaire and semistructured interview administered to participants after each session to collect feedback on their satisfaction with the VR game and suggestions for improvements (Multimedia Appendix 1).

Each session involved the presence of an expert observer positioned in front of the users, whose task was to record the behaviors exhibited by the participants during the VR activity. At the end of each session, the observer and therapist would jointly fill out a specially prepared observation form, recording the observed behaviors.

In addition, each session was video recorded, which allowed for the review of the sessions and further coding of the participants’ behavior.

### Ethics Approval

The study was approved by the research ethics committee of Azienda Provinciale Servizi Sanitari, Trento on March 15, 2023 (report 4959).

## Results

### Social Skills Questionnaire

A comparative analysis of the pre- and postintervention SSQ responses (Teacher form) for matched participants was performed using the Wilcoxon signed rank test. This test was used to examine the statistical significance of the improvement in social and communication competence of the participants based on the aggregated data of the 12 participants. Wilcoxon signed rank tests determined that there was a statistically significant median increase (*W*=1, *P*=.008; critical value of *W*=1 at n=8) between pre- and postintervention scores, indicating that the participants showed significantly higher frequencies of positive social interaction and communication competence after the intervention.

### Postsession Observations

Postsession observations by the therapist were mainly oriented toward understanding the behaviors exhibited by the participants during the VR game sessions to identify possible usability issues. Different behaviors were considered to gather information about the user experience with the VR game, including (1) qualitative measures of participants’ adaptation and engagement with the game, (2) a systematic evaluation of participants’ interactions and communication, and (3) an assessment of participants’ emotional states during the activities.

Overall, all the participants displayed a natural curiosity and interest in the experience, which further grew over time. This observation was supported by various qualitative indicators, including positive and enthusiastic expressions (both verbal and nonverbal) during the tasks as well as during active engagement.

**Figure 1:**
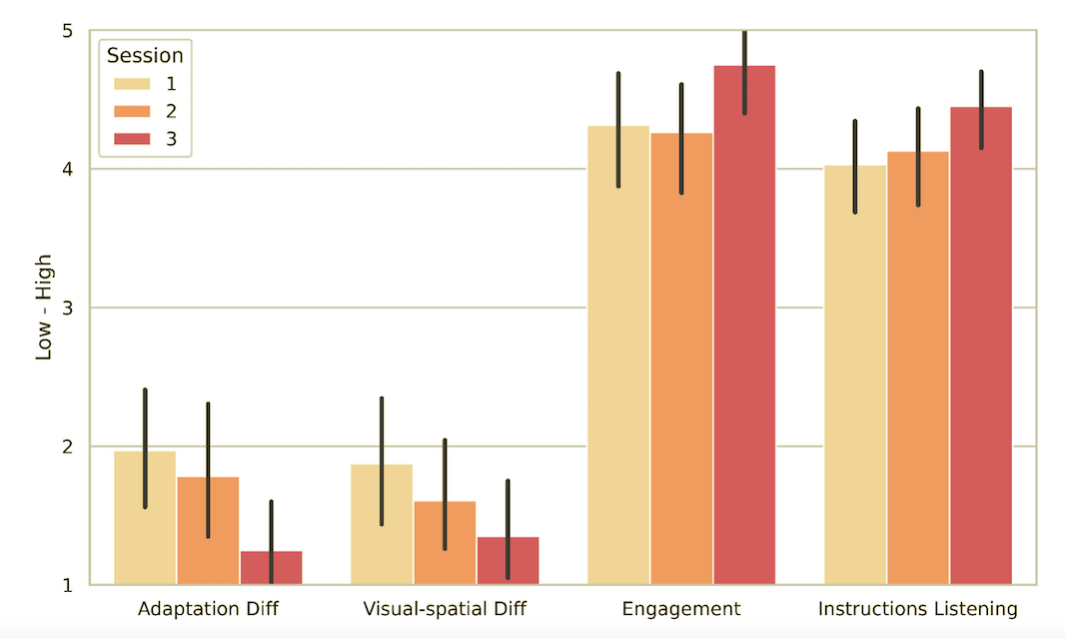
Participants’ behavioral assessment (first part) during the virtual reality (VR) gaming missions, expressed on a scale of 1 to 5 (1=low; 5=high).

During the game sessions, most participants actively shared their improvements, difficulties, impressions, and questions with other players and, particularly, with their facilitators. The collaborative interactions among the participants increased as the game missions progressed (Figure 1; Table 4).

We observed a growth in self-confidence among the participants throughout the game missions, as suggested by a gradual increase in the level of engagement with the activities as well as a decrease in adaptive distress in VR scenarios and visuospatial difficulties when interacting with the virtual surroundings (Figure 1; Table 4). Initially, they primarily focused on following the game missions and did not fully explore the virtual environment. However, gradually, they started venturing into the game scenario, comprehending its dynamics and, most importantly, feeling increasingly capable and secure.

The first mission (Coin Hunt) emphasized collaboration and communication among the participants, whereas the third mission (Planet Battle) actively engaged the participants to a greater extent than other missions. It is possible that these results were influenced by the game dynamics. Coin Hunt offered a lesser challenging and emotionally impactful setting than other missions. Thus, this environment might have facilitated increased social interactions among participants as they worked together to complete the task. By contrast, according to participant reports, Planet Battle was the most enjoyable game mission, which increased participants’ motivation and engagement.

Overall, the participants successfully completed all game sessions, consistently achieving high scores. Notably, their game scores showed a clear upward trend across the sessions, even if the difficulty level was progressively increased by the facilitators.

**Table 4:** Participants’ behavioral assessment (first part) during the virtual reality (VR) gaming missions, expressed on a scale of 1 to 5 (1=low; 5=high).

Feature	First mission, mean score (SD)	Second mission, mean score (SD)	Third mission, mean score (SD)
Difficulties in adapting to VR	2.0 (1.3)	1.7 (1.2)	1.3 (0.7)
Visuo-motor difficulties	1.9 (1.3)	1.6 (1.0)	1.4 (0.8)
Listening to instructions	4.0 (1.0)	4.1 (0.9)	4.5 (0.6)
Engagement with the game	4.3 (1.2)	4.3 (1.0)	4.8 (0.7)

In addition, to obtain a more systematic measurement of the social patterns displayed during the VR activities, we quantified the number of social exchanges. The group social behaviors considered were social initiations and social responses to peers. Moreover, we differentiated between spontaneous social exchanges and prompted social exchanges. Spontaneous social exchanges included initiations and responses manifested spontaneously by the participant toward peers. By contrast, prompted exchanges included initiations or responses that were not spontaneously manifested by the participant but were solicited by the therapist through verbal cues. This differentiation allowed us to distinguish social exchanges based on spontaneity and to analyze any differences between activities. Furthermore, we noted the number of times each participant requested a break during the game. We quantitatively compared the distributions of these parameters among the 3 game missions using the Kruskal-Wallis test. Our results indicated a significant difference in both spontaneous (H_2_=49.6, *P*<.001) and prompted (H_2_=14.8, *P*<.001) social initiations and spontaneous (H_2_=43.3, *P*<.001) and prompted (H_2_=11.6, *P*=.003) social responses, with the first mission exhibiting higher scores than the subsequent ones. In addition, the number of requested breaks was significantly higher in the first mission, confirming that Coin Hunt is the most socially promoting activity. The collaborative and communicative nature of the gameplay is reflected in the higher number of social initiations and responses observed during this mission. Moreover, a higher need for breaks during this game mission by users corroborates this analysis, highlighting the critical role of appropriate social interaction modes for individuals with ASD. To gain a broader perspective on the nature of these results, we compared the ratio between spontaneous and prompted social exchanges. Our analysis of the distribution of spontaneous and prompted initiations and responses using the Mann-Whitney test revealed that the participants showed a significantly greater number of spontaneous social openings (mean 12.9, SD 11.4) than prompted social openings (mean 0.7, SD 1.2; *U*=76.0, *P*<.001) and a higher number of spontaneous social responses (mean 11.8, SD 0.6) than prompted social responses (mean 0.3, SD 0.6) to peers (*U*=74.5, *P*<.001), emphasizing the effectiveness of the game missions in promoting spontaneous group interactions.

To obtain a more systematic measurement regarding the participants’ emotional state, we classified the gaming sessions by qualitatively indicating the nature of the activation state during the VR experience. The emotional state was encoded in agreement with 3 independent evaluators using 2 distinct parameters. The first parameter coded the level of activation and was characterized as calm, agitated, or dysregulated. The second parameter referred to the level of participation in the activity and was classified as active or passive. The results indicated that 75% (9/12) of the players approached the activity calmly, while only 25% (3/12) manifested episodes of agitation, and none of the participants became dysregulated during the activities. Moreover, >86% (10/12) of the players participated actively in the scenarios, whereas only 14% (2/12) showed a passive attitude. Overall, the participants maintained a positive attitude during the game sessions, demonstrating collaboration and maintaining a high level of attention and activity. Moreover, the analysis showed a low level of frustration experienced by most participants. We observed 9 episodes of frustration during the first mission (mean 1.6, SD 0.5), 2 episodes during the second mission (mean 1.5, SD 0.5), and 1 episode during the last mission. The observed results could indeed be attributed to increased participant familiarity and adaptation throughout the different missions, both in terms of knowledge of the VR game and the ability to use the controllers. As participants progressed through the game missions, they likely became more comfortable with the VR environment and developed better skills in using the controllers. This increased familiarity and skill level may have contributed to the decrease in frustration episodes during subsequent missions. These results confirmed that the VR game provides a controlled and safe environment that supports skills training.

### Results of the Interviews With the Participants

The interviews with participants at the end of the VR gaming experience were mainly oriented toward evaluating whether the VR gaming experience met the participants’ expectations and identifying possible usability issues. Different questions were used to gather information about user experience with the VR game. The results from the rating on a Likert scale (1=not at all; 5=very much) showed an overall appreciation of the VR game user experience by all participants (12/12, 100%), who found the VR game easy to use, clear, and engaging (Table 5), with no participants reporting interaction issues or discomfort with the VR game (Table 6).

To further confirm this positive experience of participants with the VR game, we asked them to express their willingness to play again with it after the end of the intervention (Table 4). All the participants (12/12, 100%) were willing to play again, and 92% (11/12) of them reported experiencing positive feelings, such as happiness and relaxation, during the VR gaming session and at the end of the intervention.

**Table 5:** Participants’ appreciation of the virtual reality (VR) gaming environment on a scale of 1 to 5 (1=not at all; 5=very much) in the feasibility study.

Factor measured	Score, mean (SD)
Clarity of VR game	4.5 (0.5)
Engagement	4.8 (0.3)
Interaction with objects	4.3 (1.1)
Movement in VR	4.9 (0.2)
Object grasping	4.6 (0.4)
Interaction with the environment	4.7 (0.4)
Focus on activity	4.8 (0.3)
Enjoyment	4.7 (0.4)
Willingness to play again	4.8 (0.3)

**Table 6:** Participants’ difficulties experienced with the virtual reality gaming environment on a scale of 1 to 5 (1=not at all; 5=very much) in the feasibility study.

Factor measured	Score, mean (SD)
Difficulty with Oculus headset	1.1 (1.5)
Difficulties with controllers	1.3 (0.6)
Nausea	1.0 (0.2)

## Discussion

### Principal Findings

The feasibility study with 12 adolescents with ASD showed a good level of acceptance of the VR game intervention, and several targeted behaviors related to social interaction were observed. Although the findings of this study have an exploratory nature, they can help guide the future design of VR game interventions that foster social skills training in adolescents with ASD. In particular, as shown in previous work [19] the design of a cooperative model for the game mechanics helped to sustain the motivation and engagement of players over the VR gaming sessions and to promote the triggering of social behaviors. This finding is also aligned with the recent studies highlighting the social motivation explanation of ASD [29], such as the importance of learners’ motivation or agency in social engagement and interaction performance. An implication is that VR-based training activities differ in their social affordance and should be selected and designed in alignment with the learner and learning needs to promote a comprehensive set of social skills [16]. In accordance with previous studies [30-32], the findings from our observations during the game sessions highlight the significance of maintaining a balance between serious content and game elements in serious games designed for individuals with ASD. Moreover, our positive findings in terms of participants’ success in completing the game missions and achieving their objectives suggest a potential alignment between the participants’ skill levels and the challenges presented in the game; this indicates a promising match between their abilities and the game’s demands [33]. Overall, we can confirm that the deployment of an inclusive co-design method during the Zentastic game development facilitated a successful merging of therapists’ knowledge and expertise with adolescents’ requirements and preferences for VR gameplay, ensuring their motivation to engage with the game over multiple sessions to train their social skills in a positive and safe VR environment.

### Limitations

This study contributes new knowledge to the inclusive co-design of VR gaming solutions for the training of adolescents with special needs; this domain of design and research is still understudied, often omitting to include the views of the key stakeholders involved in the adoption of such solutions. However, some limitations are still present.

First, the sample of participants involved in the iterative design process was rather limited, which implies that the results of our feasibility study should be interpreted with caution. Future applied research to study the effectiveness of VR gaming environments for training social skills should involve a larger sample of target users and control groups with a systematic research design. In addition, the number of female participants involved was very small, and this might have affected our design, which better represented the preferences and interests of male adolescents.

Another limitation of the feasibility study conducted is the lack of control for history effects that might be coincidental with the intervention deployment, so we cannot derive final conclusions in terms of social skills training outcomes determined by our VR game intervention. However, our results can inform future research that is needed to further assess the transferability of the social skills trained in the VR game to everyday settings, as well as the long-term deployment of the VR game intervention in natural, formal, and informal learning environments, including, for example, remote access to the VR game from home. Another interesting question to be addressed by our own and future design-based research is regarding the effect of different design and implementation features of VR game interventions for training social skills of adolescents with ASD, so that we can support their long-term training in VR with a more diversified range of missions targeting additional behavioral skills.

### Conclusions

Overall, the results of our feasibility study showed that playing the Zentastic multiplayer VR game had a positive effect on supporting the enhancement of social skills and competence in adolescents with ASD, as evidenced by the results of the SSQ scores before and after intervention. Data collected from the postsession observations by therapists and from the interviews with participants further confirmed the value of the VR game in terms of user engagement, safety, and well-being during the training activities in VR; this would encourage the future application of VR gaming solutions for the training of adolescents with ASD and, potentially, other neurodevelopmental disorders. Even if further research is necessary to validate these results, these initial findings could be considered as promising indications of the usefulness of adopting an inclusive co-design approach when developing VR gaming solutions for the training of skills in this target population.

## Appendix

Multimedia Appendix 1 Zentastic virtual reality game interview.

## Abbreviations

ASD: autism spectrum disorder
SSQ: Social Skills Questionnaire
VR: virtual reality
