# Supplementary material for: Co-Design of a Virtual Reality Multiplayer Adventure Game for Adolescents With Autism Spectrum Disorder: Mixed Methods Study
Source: JMIR Serious Games. 2023 Dec 8;11:e51719. doi: 10.2196/51719 (PMC10746967; doi:10.2196/51719)
Supplement: Multimedia Appendix 1 [file games_v11i1e51719_app1.pdf]

Zentastic VR Game Semi-Structured Interview

Participant ID:______ Date: __________ Interviewer:__________
Participant age:______
Experience with VR: Y N
Experience with videogames: Y N

Interview Questions

Evaluation: Likert scale 1= Not at all 5= Very much:

1. *Was the functioning of the virtual scenario clear?*

1	2	3	4	5
Not at all				*Very much*

2. *Did you have problems with the Oculus headset, was it annoying?*

1	2	3	4	5
Not at all				*Very much*

3. *Did you find difficult to learn how to use the joystick?*

1	2	3	4	5
Not at all				*Very much*

4. *Did you find engaging the visual elements of the VR (were they clear enough)?*

1	2	3	4	5
Not at all				*Very much*

5. *Did you manage to interact with the objects available in the VR?*

1	2	3	4	5
Not at all				*Very much*

6. *Did you manage to move easily within the VR game?*

1	2	3	4	5
Not at all				*Very much*

7. *How much did you manage to grasp/move the objects in the VR game?*

1	2	3	4	5
Not at all				*Very much*

8. *At the end of the session, how much did you feel able to move and interact with the VR?*

1	2	3	4	5
Not at all				*Very much*

9. *How much did you feel able to concentrate in the activities rather than on the mechanism for performing those activities?*

1	2	3	4	5
Not at all				*Very much*

10. *Did you experience any nausea/headache during the experience in VR?*

1	2	3	4	5
Not at all				*Very much*

11. *Did you like the experience in the augmented reality? Why?*

1	2	3	4	5
Not at all				*Very much*

12. What did you dislike in the experience in VR? Why?

____

____

____

13. Would you play again in VR?

1	2	3	4	5
Not at all				*Very much*

14. How did you feel during your experience in VR?

Happy Sad Scared Annoyed Relaxed Angry Dazed Confused

15. How do you feel now?

Happy Sad Scared Annoyed Relaxed Angry Dazed Confused

16. Any additional observation?

____

____
